# Risk of Placental Pathology Across Ultrasound‐Defined Phenotypes of Impaired Fetal Growth in Dichorionic Twins: A Retrospective Cohort Study

**DOI:** 10.1111/1471-0528.70215

**Published:** 2026-03-11

**Authors:** Ala Essalah, John Kingdom, Liran Hiersch, Jon Barrett, Megan Watson, Nir Melamed

**Affiliations:** ^1^ Division of Maternal‐Fetal Medicine, Department of Obstetrics and Gynaecology, Sunnybrook Health Sciences Center, Temerty Faculty of Medicine University of Toronto Toronto Ontario Canada; ^2^ Division of Maternal‐Fetal Medicine, Department of Obstetrics and Gynaecology, Mount Sinai Hospital, Temerty Faculty of Medicine University of Toronto Toronto Ontario Canada; ^3^ Lis Maternity Hospital, Sourasky Medical Center, and Gray Faculty of Medical and Health Science Tel Aviv University Tel Aviv Israel; ^4^ Department of Obstetrics and Gynaecology McMaster University Hamilton Ontario Canada

**Keywords:** discordance, discordant, doppler, FGR, growth charts, growth restriction, histopathology, IUGR, SGA, twin

## Abstract

**Objective:**

To determine the risk of placental dysfunction associated with different ultrasound‐based phenotypes of impaired fetal growth in dichorionic twin pregnancies.

**Design:**

Retrospective cohort study.

**Setting:**

Single tertiary centre.

**Population:**

Patients with dichorionic twin pregnancies delivered at ≥ 32^0/7^ (2011–2023) (*N* = 753).

**Methods:**

Each twin fetus was classified into a distinct exposure group based on combinations of fetal weight percentile (categorised as AGA, mild SGA, or severe SGA) based on singleton‐ or twin‐specific growth chart, weight discordance and Doppler.

**Main Outcome Measures:**

Abnormal placental histopathology known to be associated with FGR.

**Results:**

The rate of the primary outcome in the reference group (AGA, concordant growth, normal Doppler) was 12.8%. When using a singleton chart, isolated discordance and mild SGA were not associated with the primary outcome. Only isolated severe SGA (28.8%, aRR 2.47[1.52–4.03]), followed by severe SGA with discordant growth (41.9%, aRR 3.53[2.15–5.79]) and abnormal Doppler (54.7%, aRR 4.33[2.90–6.48]) showed significant associations with the primary outcome. In contrast, when using a twin‐specific chart, both isolated discordance (32.4%, aRR 2.63[1.46–4.73]) and mild SGA (28.2%, aRR 2.43[1.30–4.51]) were associated with the primary outcome. We propose three risk categories for late‐onset placenta‐mediated FGR based on the likelihood of the primary outcome: (1) possible small increase (< 2‐fold); (2) moderate increase (2–3 fold); and (3) highest risk (> 4‐fold).

**Conclusion:**

The risk categories proposed in the current study can serve as a framework to inform the design of future trials and contribute to the development of guidelines for managing twin pregnancies affected by late‐onset FGR.

## 1 Introduction

Fetal growth restriction (FGR), defined as the failure of a fetus to achieve its growth potential [1], is a leading cause of perinatal mortality and morbidity [2, 3, 4, 5, 6, 7, 8, 9, 10]. Twin pregnancies, accounting for approximately 3% of births [11, 12], are at increased risk of FGR and associated adverse perinatal outcomes compared to singleton pregnancies [13, 14, 15, 16, 17]. However, several unique factors complicate the diagnosis of FGR in twin pregnancies. First, it remains unclear whether the slower growth rate of twins during the third trimester represents a pathological placental insufficiency or a benign adaptation to the competing uterine environment [17, 18, 19, 20, 21, 22]. Consequently, there is an ongoing debate over the fetal weight percentile cut‐offs that should be used to diagnose FGR and whether the diagnosis should be based on singleton or twin‐specific growth charts [18, 23, 24, 25, 26]. Second, unlike singleton pregnancies, the growth of the twin fetus can be evaluated not only against published reference growth charts but also in relation to its co‐twin, as reflected by intertwin weight discordance [27, 28, 29, 30, 31, 32, 33, 34, 35, 36, 37, 38, 39, 40].

The numerous possible combinations of the above‐mentioned sonographic measures (fetal weight percentile according to singleton‐ vs. twin‐specific charts and intertwin size discordance), along with other indicators of placenta‐mediated FGR (such as umbilical artery and middle cerebral artery Doppler), make it difficult to standardise the diagnosis and management of FGR in twin pregnancies. Thus, for example, clinicians are often unsure regarding the risks and optimal management of twin pregnancies with a fetus that is mildly small (3rd–9th percentile) or with isolated weight discordance, and whether the presence of discordant growth modifies the risk in cases with foetuses who are severely small (< 3rd percentile) or abnormal Doppler studies. This uncertainty is particularly relevant for twin pregnancies with suspected late‐onset FGR. Current guidelines recommend relatively early delivery for all twins, regardless of FGR [41, 42, 43, 44, 45], and it remains unclear whether twin foetuses with certain combinations of the clinical indicators of impaired fetal growth described above might benefit from an even earlier delivery.

A practical strategy to address this problem is to assess the risk of placenta‐mediated FGR associated with specific combinations of these various indicators of impaired fetal growth. Grouping these ultrasound‐based phenotypes into clinically meaningful categories based on the risk of underlying placental disease may help standardise fetal surveillance and timing of delivery. Additionally, this information could facilitate the design of future randomised trials on the management of twin pregnancies with FGR by standardising patient selection.

The objective of this retrospective study was to determine the risk of placental dysfunction associated with different combinations of fetal weight percentile (according to singleton and twin‐specific charts), intertwin weight discordance, and Doppler findings in dichorionic twin pregnancies. Based on these findings, we aimed to group these ultrasound‐based phenotypes of impaired fetal growth into clinically useful categories that reflect an incremental risk of placenta‐mediated FGR.

## 2 Materials and Methods

### 2.1 Study Population

We conducted a retrospective cohort study of patients with dichorionic twin pregnancies who received care and delivered at ≥ 32^0/7^ weeks’ gestation at a single tertiary referral center (Sunnybrook Health Sciences Centre, Toronto, Ontario, Canada) between January 2011 and December 2023.

Pregnancies complicated by any of the following conditions were excluded: (1) fetal genetic or structural anomalies; (2) fetal reduction of one or both twins; (3) stillbirth of one or both twins due to non‐placental etiologies (i.e., etiologies other than FGR or abruption); (4) last sonographic assessment of fetal biometry performed > 14 days before delivery; (5) missing or incomplete data on the ultrasound exam, gestational age at birth, birthweight, or placental pathology. The study was approved by the Sunnybrook Health Sciences Centre Research Ethics Board (#353–2014). Patients were not involved in the development of the current study.

### 2.2 Data Collection

Cases were identified from the obstetrical ultrasound unit database. The last ultrasound exam of potential cases was reviewed for information on the number of fetuses, gestational age at the time of the exam, amniotic fluid level, Doppler studies, and fetal biometry, including biparietal diameter, head circumference, abdominal circumference, and femur length. Estimated fetal weight (EFW) was calculated using the Hadlock 1985 formula that incorporates all four biometric indices [46]. Intertwin weight discordance was calculated as the absolute weight difference between the twins, divided by the weight of the larger twin, and expressed as a percentage: *Discordance (%) =* |*weight*_*A*_ *—weight*_*B*_|*/[weight of larger twin] x100*. Maternal characteristics, pregnancy complications, neonatal outcomes, and placental histopathology were abstracted from the medical records of the eligible cases.

According to our departmental protocol, umbilical artery Doppler studies were routinely acquired during ultrasound examinations conducted for fetal growth assessment or biophysical profile. Umbilical artery Doppler was measured in a free loop of the umbilical cord, and the results were recorded either as the pulsatility index or qualitatively as absent or reversed end diastolic flow. Middle cerebral artery Doppler was performed when FGR was suspected based on an EFW below the 10th percentile. It was obtained at the proximal third of the vessel, identified using colour Doppler, to derive the pulsatility index. The cerebroplacental ratio was calculated as the ratio of the middle cerebral artery pulsatility index to the umbilical artery pulsatility index [47].

### 2.3 Exposures

The following indicators of impaired fetal growth were assessed based on the last ultrasound examination before birth: (1) EFW percentile, determined using both singleton [48] and twin‐specific [17] ultrasound‐based fetal growth charts, and categorised as severe small for gestational age (SGA, < 3rd percentile), mild SGA (3‐9th percentile), or appropriate for gestational age (AGA, ≥ 10th percentile); (2) intertwin fetal weight discordance, classified dichotomously as discordant (> 20%) or concordant (≤ 20%); in twin pairs with discordant growth, only the smaller twin was considered affected by discordant growth, as discordance is typically interpreted as reflecting inadequate growth of the smaller (rather than excessive growth of the larger) twin [41, 42, 43] and (3) abnormal Doppler studies, defined as one or more of the following: (i) umbilical artery pulsatility index > 95th percentile [47] or absent or reversed end diastolic flow, (ii) middle cerebral artery pulsatility index < 5th percentile [47], or (iii) cerebroplacental ratio < 5th percentile [47].

Based on these three indicators, each fetus was then classified into one of eight exposure groups, representing mutually exclusive combinations of the indicators: (1) Group 1 (reference) – AGA, concordant, normal Doppler; (2) Group 2 (isolated discordance) – AGA, discordant, normal Doppler; (3) Group 3 (isolated mild SGA) – mild SGA, concordant, normal Doppler; (4) Group 4 (mild SGA with discordance) – mild SGA, discordant, normal Doppler; (5) Group 5 (isolated severe SGA) – severe SGA, concordant, normal Doppler; (6) Group 6 (severe SGA with discordance) – severe SGA, discordant, normal Doppler; (7) Group 7 (concordant SGA with abnormal Doppler) – SGA (mild or severe), concordant, abnormal Doppler; and (8) Group 8 (discordant SGA with abnormal Doppler) – SGA (mild or severe), discordant, abnormal Doppler.

We did not include a group of AGA foetuses with abnormal Doppler, as middle cerebral artery Doppler was only measured for SGA foetuses, and umbilical artery Doppler is unlikely to be abnormal in AGA foetuses. Additionally, we did not distinguish between mild and severe SGA in cases with abnormal Doppler, since abnormal Doppler is highly suggestive of placental insufficiency, and the associated risk is unlikely to be affected by the severity of SGA.

### 2.4 Outcomes

The primary outcome was abnormal placental histopathology, defined as the presence of at least one of three pathologies known to be associated with FGR: maternal vascular malperfusion (MVM), fetal vascular malperfusion (FVM), or chronic villitis (see definitions of the individual placental pathologies below). Although many previous studies on the diagnosis and definition of FGR have used neonatal outcomes (e.g., admission to a neonatal intensive care unit, a low 5‐min Apgar score, or neonatal morbidity) as proxies for placenta‐mediated FGR, such clinical outcomes are not specific to FGR and are often influenced by other exposures, particularly prematurity. Therefore, we chose abnormal placental pathology as the primary outcome, which we considered to be a direct and gestational‐age‐independent marker of placenta‐mediated FGR.

A secondary outcome was a composite of adverse neonatal outcomes, defined as the presence of at least one of the following: 5‐min Apgar score < 7, umbilical artery pH < 7.1, or admission to the neonatal intensive care unit.

We did not use a core outcome set in the current study.

### 2.5 Classification of Placental Findings

According to our departmental policy, all placentas of multifetal gestations were routinely sent for pathological examination during the study period. The placental assessments were performed by two experienced perinatal pathologists, who were blinded to the ultrasound findings. Our standard protocol for placental examination was described in detail elsewhere [49, 50, 51]. Briefly, within 24 h after delivery, the placenta, membranes, and cord were fixed in formalin. Macroscopic evaluation of the placenta, membranes, and umbilical cord included determination of chorionicity and amnionicity and assigning each placenta (or placental portion in the case of fused placentas) to the larger and smaller twin based on labelling of the cords at the time of birth. For each, gross parenchymal lesions or attached clots were noted, together with the number of umbilical cord vessels, placental cord insertion site (central, marginal, or velamentous), and hypercoiled or hypocoiled cord. Subsequently, at least six placental tissue samples were embedded in paraffin blocks for microscopic assessment. Samples were obtained from membranes, umbilical cord, centrally and marginally located tissue that appeared abnormal on gross examination, and up to three samples from normally appearing placental tissue. Placental abnormalities for each fetus were classified according to the criteria suggested by the 2014 Amsterdam Placental Workshop Group [52] as follows: (1) Maternal vascular malperfusion (MVM)—defined as two or more of the following: small placenta (< 10th percentile for gestational age) [53], decidual vasculopathy (atherosis and/or eosinophilic necrosis of decidual arterial walls), central (> 1 cm) full‐thickness or multi‐focal infarction, distal villous hypoplasia, accelerated villous maturation with syncytial knot formation, increased intervillous fibrin deposition, or retroplacental haemorrhage; (2) Fetal vascular malperfusion (FVM)—defined as large vessel thrombosis or fetal thrombotic vasculopathy (hypovascular, fibrotic and avascular villi); or (3) Chronic villitis—defined as villitis of unknown aetiology, chronic deciduitis, or chronic intervillositis.

### 2.6 Definitions and Protocols

All twin pregnancies in our institution are managed based on a standardised protocol. Gestational age and chorionicity are confirmed by first‐trimester ultrasound in all cases. Dichorionic twin pregnancies are monitored every 2–4 weeks. EFW percentiles are determined using the singleton‐based Hadlock growth chart [48]. Cases with suspected FGR are monitored once or twice weekly, depending on the severity of FGR and the presence of intertwin size discordance, abnormal Doppler studies, or oligohydramnios [41]. Timing of delivery follows the same recommendations as for singleton pregnancies with suspected FGR. For uncomplicated dichorionic twin pregnancies, labor induction or planned caesarean delivery is scheduled at 37^0/7^–37^6/7^ weeks of gestation [7, 41, 45].

### 2.7 Data Analysis

Standard descriptive statistics were used to summarise the baseline characteristics and outcomes of the study population.

We first employed restricted cubic splines to model the probability of the primary outcome as a function of EFW percentile (using both singleton and twin‐specific charts) and intertwin EFW discordance. For intertwin EFW discordance (and only for the purposes of this specific analysis), we considered the probability of abnormal placental pathology only for the smaller twin, since discordance is typically interpreted as reflecting inadequate growth and possible placental dysfunction in the smaller twin (rather than normal growth or absence of placental dysfunction in the larger twin). The Bayesian Information Criterion (BIC) was used to select the optimal number of knots (from 3 to 6) to ensure model parsimony [54]. Knots were placed at data‐driven threshold percentiles. This analysis was conducted using R version 4.3.3 (R Core Team, 2023). The purpose of this modelling was to confirm the appropriateness of the thresholds used to define mild SGA, severe SGA, and discordant growth.

We then calculated the rate of the study outcomes for each of the eight exposure groups. We used generalized linear models, assuming a Poisson distribution, to estimate the relative risks (RRs) with 95% confidence intervals (CIs) for the study outcomes for each group, using Group 1 (AGA, concordant, normal Doppler) as the reference. Twin pairs were treated as clusters to account for within‐pair correlation. Models were adjusted for maternal age, nulliparity, gestational age at birth, and fetal sex group, all of which were defined a priori. This analysis was conducted using SPSS version 29.0 (IBM Corp, Armonk, NY). Hypotheses were tested using two‐tailed tests with a significance level of 0.05.

## 3 Results

### 3.1 Characteristics of the Study Population

Of the 1457 dichorionic twin pregnancies identified during the study period, 753 pregnancies (1506 foetuses) met the study criteria (Figure S1). The characteristics and outcomes of the study population are summarised in Table 1. The median interval between the last ultrasound examination and birth was 6 days. The mean gestational age at birth was 36.0 ± 1.6 weeks, and 9.4% of infants were born before 34 weeks. The rate of preeclampsia was 4.5%, and the proportions of infants with birthweights below the 10th and 3rd percentiles (based on a twin‐specific growth chart) were 12.9% and 4.3%, respectively, reflecting the unselected nature of the study cohort (Table 1). The rates of the primary outcome (abnormal placental histopathology) and the secondary composite neonatal outcome were 16.8% and 19.9%, respectively (Table 1).

**TABLE 1:** Baseline characteristics and outcomes of the study cohort.

Characteristic or outcome	Value
Number of patients (pregnancies)	753
Maternal age (years), mean ± SD	34.4 ± 5.0
≥ 35 years, *n* (%)	298 (39.6%)
Nulliparity, *n* (%)	432 (57.4%)
Pre‐existing diabetes, *n* (%)	8 (1.1%)
Gestational diabetes, *n* (%)	88 (11.7%)
Chronic hypertension, *n* (%)	13 (1.7%)
In vitro fertilisation, *n* (%)	140 (18.6%)
Preeclampsia, *n* (%)	34 (4.5%)
Last ultrasound exam to birth interval (days), median (IQR)	6 (2–10)
Caesarean delivery, *n* (%)	513 (68.1%)
Gestational age at birth (weeks), mean ± SD	36.0 ± 1.6
< 37 weeks, *n* (%)	399 (53.0%)
< 34 weeks, *n* (%)	71 (9.4%)
*Fetal sex group, n (%)*	
Female–Female	205 (27.2%)
Female–Male	338 (44.9%)
Male–Male	210 (27.9%)
Birthweight discordance (%), mean ± SD	12.2% ± 10.9%
> 15%, *n* (%)	228 (30.3%)
> 20%, *n* (%)	128 (17.0%)
> 25%, *n* (%)	58 (7.7%)
Birthweight percentile (singletons chart of Hadlock)	
< 10th centile, *n* (%)^a^	434 (28.8%)
< 3rd centile, *n* (%)^a^	191 (12.7%)
Birthweight percentile (twins chart of Hiersch)	
< 10th centile, *n* (%)^a^	194 (12.9%)
< 3rd centile, *n* (%)^a^	65 (4.3%)
5‐min Apgar < 7, *n* (%)^a^	47 (3.1%)
Umbilical artery pH < 7.1, *n* (%)^a^	2 (0.1%)
NICU admission, *n* (%)^a^	271 (18.0%)
Composite adverse neonatal outcome, *n* (%)^a^ ^,^ ^b^	300 (19.9%)
Abnormal placental pathology, *n* (%)^a^ ^,^ ^c^	253 (16.8%)
Maternal vascular malperfusion (MVM) pathology, *n* (%)	144 (9.6%)
Fetal vascular malperfusion pathology (FVM), *n* (%)	84 (5.6%)
Chronic villitis, *n* (%)	65 (4.3%)

Abbreviation: NICU, neonatal intensive care unit.

^a^ The unit of analysis for this outcome is the fetus rather than pregnancy (*N* = 1506).

^b^ At least one of the following: 5‐min Apgar score < 7, umbilical artery pH < 7.1, or NICU admission.

^c^ 40 (15.8%) placentas exhibited more than one pathology diagnostic category.

### 3.2 Probability of the Primary Outcome in Relation to Fetal Weight Percentile and Intertwin Discordance

We first examined the probability of the primary outcome (abnormal placental histopathology) as a function of EFW percentile and intertwin EFW discordance (Figure S2).

For EFW percentile, the probability of abnormal placental pathology increased markedly when the percentile fell below the 10th percentile, regardless of the type of growth chart used, supporting the use of the 10th and 3rd percentiles to define mild and severe SGA, respectively, for both singleton and twin‐specific charts (Figure S2A).

For intertwin EFW discordance, the probability of abnormal placental pathology increased sharply when discordance exceeded 20%, thereby justifying the use of this threshold to define discordant growth in the classification of the FGR phenotypic groups (Figure S2B).

### 3.3 Description of the Eight Exposure Groups

Table 2 presents the definition and relative proportions of the eight combinations of indicators of impaired fetal growth. The number of fetuses in the AGA‐concordant group (Group 1, reference) was 1109 (73.6%) and 1292 (85.8%) when SGA was defined using singleton and twin‐specific charts, respectively (Table 2). The proportion of fetuses with isolated discordance (Group 2) was small (0.7% and 2.5% when SGA was defined using singleton and twin‐specific charts, respectively), indicating that in most discordant twin pairs, the smaller twin is classified as SGA.

**TABLE 2:** Description of the eight exposure groups.

Exposure group	Criteria			Number of foetuses per group (*N* = 1506)	
	EFW centile	EFW discordance ≥ 20%^a^	Abnormal doppler^b^	Using singleton charts^c^ [*n* (%)]	Using twin charts [*n* (%)]^d^
G1: AGA‐Concordant (reference)	≥ 10th			1109 (73.6%)	1292 (85.8%)
G2: AGA‐discordant	≥ 10th	—		10 (0.7%)	37 (2.5%)
G3: Mild SGA‐concordant	3–9th			169 (11.2%)	39 (2.6%)
G4: Mild SGA‐discordant	3–9th	—		23 (1.5%)	20 (1.3%)
G5: Severe SGA‐concordant	< 3rd			66 (4.4%)	13 (0.9%)
G6: Severe SGA‐discordant	< 3rd	—		43 (2.9%)	19 (1.3%)
G7: SGA‐Abn doppler—concordant	< 10th		—	53 (3.5%)	53 (3.5%)
G8: SGA‐Abn doppler—discordant	< 10th	—	—	33 (2.2%)	33 (2.2%)

Abbreviations: AGA, appropriate for gestational age; EFW, estimated fetal weight; FGR, fetal growth restriction; SGA, small for gestational age.

^a^ In twin pairs with discordant growth, only the smaller twin within that pair was considered to be affected by growth discordance.

^b^ Refers to abnormal umbilical artery Doppler, middle cerebral artery Doppler, or cerebroplacental ratio.

^c^ Based on the singleton chart of Hadlock et al.

^d^ Based on the twin‐specific chart of Hiersch et al.

### 3.4 Association of the Eight Exposure Groups With Abnormal Placental Pathology

**FIGURE 1:**
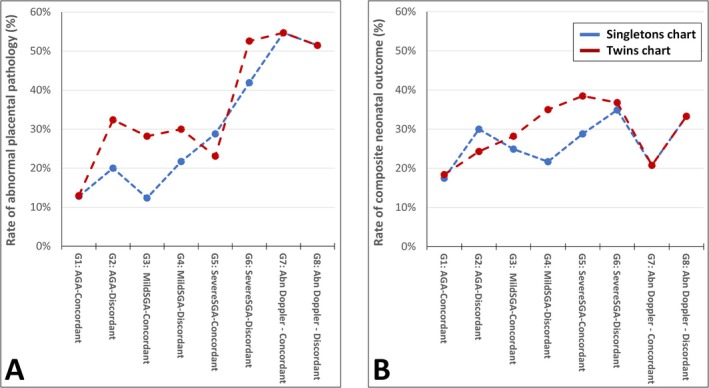
Rate of the study outcomes by the exposure group. The rates of the primary outcome (abnormal placental histopathology) (A) and the secondary composite neonatal outcome (B) are presented for each phenotype of impaired fetal growth, defined using either a singleton chart (blue line) or a twin‐specific chart (red line). AGA, appropriate for gestational age; SGA, small for gestational age.

**FIGURE 2:**
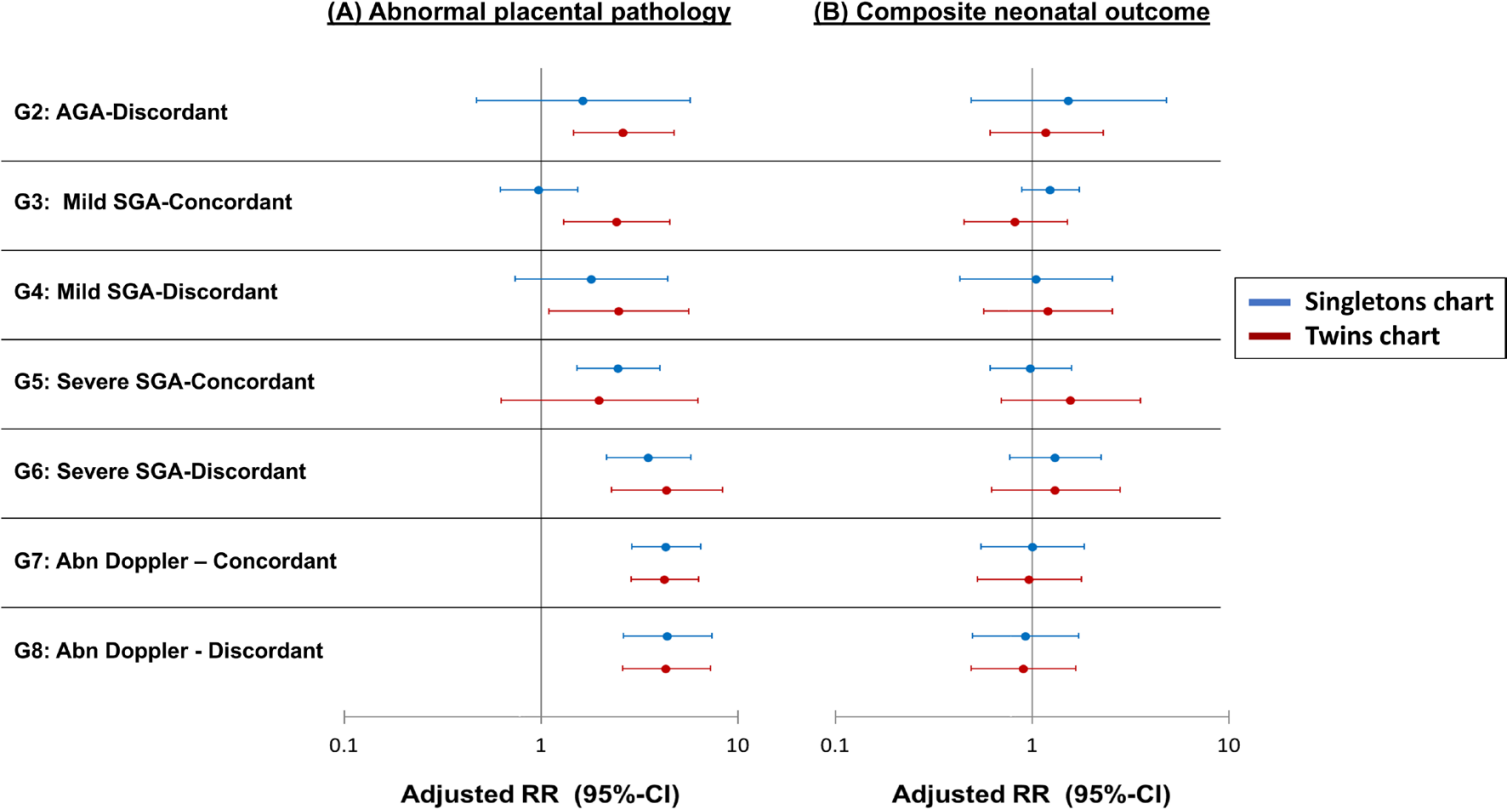
Association of the exposure groups with the study outcomes. The figure presents the association between the combinations of indicators of impaired fetal growth and the primary outcome of abnormal placental pathology (A) and the secondary composite neonatal outcome (B) Fetal weight percentiles were defined using either a singleton chart (blue lines) or a twin‐specific chart (red lines). Associations were estimated using generalized linear models, assuming a Poisson distribution, and are presented as relative risk (RR) with 95% confidence intervals (95% CI) using Group 1 (AGA, concordant, normal Doppler) as the reference. Models were adjusted for maternal age, nulliparity, gestational age at birth, and fetal sex group. AGA, appropriate for gestational age; GA, small for gestational age.

The unadjusted rates of the primary outcome in each of the combinations of indicators of impaired fetal growth are presented in Figure 1A. When using a singleton growth chart to define SGA, the rate of abnormal placental pathology in cases of isolated discordance (Group 2) and mild SGA (Groups 3 and 4) was relatively similar to that of the AGA‐concordant reference group (Group 1), remaining in the range of 12%–21%. The rate began to increase only in cases of severe SGA with concordant growth (28.8%), rising further in cases of severe SGA with discordant growth (41.9%) and was highest in cases with abnormal Doppler studies, regardless of the co‐presence of discordant growth (51.5%–54.7%) (Figure 1A). A similar pattern was observed in the adjusted analysis (Table 3 and Figure 2). Neither isolated discordance nor mild SGA (regardless of discordance) was significantly associated with the primary outcome. Only isolated severe SGA was associated with a ~2.5‐fold increased risk of the primary outcome, followed by severe SGA with discordant growth (~3.5‐fold increased risk), and abnormal Doppler (> 4‐fold increased risk).

**TABLE 3:** Risk of the study outcomes in each of the exposure groups.

Exposure group	Risk of outcome—singleton chart			Risk of outcome—twin chart		
	Rate [*n* (%)]	Crude RR (95% CI)	Adjusted RR (95% CI)^a^	Rate [*n* (%)]	Crude RR (95% CI)	Adjusted RR (95% CI)^a^
Primary outcome: Abnormal placental pathology						
G1: AGA‐Concordant	142 (12.8%)	Reference	Reference	168 (13.0%)	Reference	Reference
G2: AGA‐Discordant	2 (20.0%)	1.56 (0.45–5.45)	1.64 (0.47–5.72)	12 (32.4%)	**2.49 (1.39–4.48)**	**2.63 (1.46–4.73)**
G3: Mild SGA‐concordant	21 (12.4%)	0.97 (0.61–1.54)	0.98 (0.62–1.54)	11 (28.2%)	**2.17 (1.18–3.99)**	**2.43 (1.30–4.51)**
G4: Mild SGA‐discordant	5 (21.7%)	1.70 (0.70–4.14)	1.81 (0.74–4.41)	6 (30.0%)	**2.31 (1.02–5.21)**	**2.49 (1.10–5.62)**
G5: Severe SGA‐concordant	19 (28.8%)	**2.25 (1.39–3.63)**	**2.47 (1.52–4.03)**	3 (23.1%)	1.78 (0.57–5.56)	1.99 (0.63–6.28)
G6: Severe SGA‐discordant	18 (41.9%)	**3.27 (2.00–5.34)**	**3.53 (2.15–5.79)**	10 (52.6%)	**4.05 (2.14–7.66)**	**4.37 (2.29–8.33)**
G7: Abn Doppler—concordant	29 (54.7%)	**4.27 (2.87–6.37)**	**4.33 (2.90–6.48)**	29 (54.7%)	**4.21 (2.84–6.24)**	**4.27 (2.87–6.34)**
G8: Abn Doppler—discordant	17 (51.5%)	**4.02 (2.43–6.65)**	**4.40 (2.62–7.40)**	17 (51.5%)	**3.96 (2.41–6.52)**	**4.35 (2.60–7.27)**
Secondary outcome: composite neonatal outcome						
G1: AGA‐concordant	194 (17.5%)	Reference	Reference	238 (18.4%)	Reference	Reference
G2: AGA‐discordant	3 (30.0%)	1.72 (0.55–5.36)	1.54 (0.49–4.83)	9 (24.3%)	1.32 (0.68–2.57)	1.18 (0.61–2.30)
G3: Mild SGA‐concordant	42 (24.9%)	1.42 (1.02–1.98)	1.24 (0.89–1.74)	11 (28.2%)	1.53 (0.84–2.80)	0.82 (0.45–1.51)
G4: Mild SGA‐discordant	5 (21.7%)	1.24 (0.51–3.02)	1.05 (0.43–2.56)	7 (35.0%)	1.90 (0.90–4.03)	1.21 (0.57–2.57)
G5: Severe SGA‐concordant	19 (28.8%)	1.65 (1.03–2.64)	0.99 (0.61–1.59)	5 (38.5%)	**2.09 (1.04–4.20)**	1.58 (0.70–3.55)
G6: Severe SGA‐discordant	15 (34.9%)	1.99 (1.18–3.37)	1.32 (0.77–2.24)	7 (36.8%)	2.00 (0.94–4.24)	1.32 (0.62–2.81)
G7: Abn Doppler—concordant	11 (20.8%)	1.19 (0.65–2.18)	1.01 (0.55–1.85)	11 (20.8%)	1.13 (0.62–2.06)	0.97 (0.53–1.78)
G8: Abn Doppler—discordant	11 (33.3%)	1.91 (1.04–3.50)	0.93 (0.50–1.73)	11 (33.3%)	1.81 (0.99–3.31)	0.91 (0.49–1.67)

*Note:* Significant associations are emphasised in bold font.

Abbreviations: AGA, appropriate for gestational age; CI, confidence interval; FGR, fetal growth restriction; RR, relative risk; GA, small for gestational age.

^a^ Associations were estimated using generalised linear models, assuming a Poisson distribution, and are presented as relative risk (RR) with 95% confidence intervals (95% CI) using Group 1 (AGA, concordant, normal Doppler) as the reference. Models were adjusted for maternal age, nulliparity, gestational age at birth, and fetal sex group.

In contrast, when using a twin‐specific growth chart, both isolated discordance and mild SGA were associated with a higher rate of the primary outcome compared with the AGA‐concordant reference group (28.2%–32.4% vs. 13.0%, respectively) (Figure 1A). The highest rates were again observed in cases of severe SGA with discordant growth (52.6%) and cases of abnormal Doppler (51.5%–54.7%). The same pattern was observed in the adjusted analysis (Table 3 and Figure 2): isolated discordance and mild SGA (regardless of discordance) were associated with a 2–3‐fold increased risk, while severe SGA with discordant growth and abnormal Doppler were associated with a > 4‐fold increased risk of abnormal placental pathology (Table 3 and Figure 2).

The rates of the individual placental pathology types were 9.6% for MVM, 5.6% for FVM, and 4.3% for chronic villitis (Table 1). The unadjusted rates of each placental pathology type in each of the combinations of indicators of impaired fetal growth are presented in Figure S3.

### 3.5 Association of the Eight Exposure Groups With the Composite Neonatal Outcome

The unadjusted rates of the secondary composite neonatal outcome across the eight exposure groups are presented in Figure 1B. Overall, the variation in outcome rates between groups was relatively modest, ranging from 17.5% to 38.5%, with no consistent relationship observed between the severity of the combination of indicators of impaired fetal growth and the rate of the composite outcome (Figure 1B). In the adjusted analysis, none of the groups demonstrated a statistically significant association with the composite neonatal outcome, especially when the models were adjusted for gestational age at birth (Table 3 and Figure 2).

### 3.6 Proposed Risk Categories for Late‐Onset FGR in Dichorionic Twin Pregnancies

Based on the observed associations with the primary outcome, we identified three risk categories for late‐onset placenta‐mediated FGR in twin pregnancies (Table 4): (1) Category 1, associated with a possible small increase (< 2‐fold) in the risk of placenta‐mediated FGR; (2) Category 2, associated with a moderate increase (2‐ to 3‐fold); and (3) Category 3, associated with the highest risk (> 4‐fold).

**TABLE 4:** Proposed risk categories for late‐onset FGR in twin pregnancies.

Combination of indicators of impaired fetal growth	Risk category when using singleton charts	Risk category when using twin charts
G1: AGA‐concordant	Category 0 (reference)	Stage 0 (reference)
G2: AGA‐discordant	Category 1 (< 2‐fold risk)	Category 2 (2–3 fold risk)
G3: Mild SGA‐concordant		
G4: Mild SGA‐discordant		
G5: Severe SGA‐concordant	Category 2 (2–3 fold risk)	
G6: Severe SGA‐discordant		Category 3 (> 4‐fold risk)
G7: Abn Doppler—concordant	Category 3 (> 4‐fold risk)	
G8: Abn Doppler—discordant		

The colour coding for the risk categories in increasing order of severity: Category 0 = gray, Category 1 = Green, Category 2 = Orange, Category 3 = Red.

When using a singleton growth chart, Category 1 comprised cases of isolated discordance or mild SGA, Category 2 comprised severe SGA with normal Doppler, and Category 3 comprised cases with abnormal Doppler findings. In contrast, when using a twin‐specific growth chart to define SGA, even cases of isolated discordance or mild SGA are associated with a 2–3‐fold increase in the risk of FGR and are therefore classified as Category 2, along with severe SGA with concordant growth, while Category 3 comprised cases of severe SGA with discordant growth and cases with abnormal Doppler findings (Table 4).

## 4 Discussion

### 4.1 Principal Study Findings

In the current study, our objective was to determine the risk of placental dysfunction associated with different phenotypes of impaired fetal growth in dichorionic twin pregnancies and group these phenotypes into clinically useful risk categories. Our main findings were as follows: (1) When a singleton growth chart was used, the antenatal diagnosis of isolated discordance or mild SGA did not increase the risk of placenta‐mediated FGR; (2) In contrast, when using a twin‐specific growth chart, the antenatal diagnosis of isolated discordance or mild SGA was associated with a significantly increased risk of placenta‐mediated FGR that was similar to that observed with the diagnosis of severe SGA based on a singleton growth chart; (3) The highest risk of underlying placental disease was observed in cases of severe SGA with discordant growth based on a twin‐specific growth chart and in the presence of abnormal Doppler studies, regardless of the chart type or coexisting discordance; (4) Based on these findings we identified three risk categories reflecting an incremental likelihood of late‐onset placenta‐mediated FGR in dichorionic twin pregnancies; and (5) In contrast to their association with abnormal placental pathology, the antenatal diagnosis of SGA, discordant growth and abnormal Doppler were not associated with the composite neonatal outcome.

### 4.2 Results in the Context of Previous Observations

One of the main challenges in diagnosing FGR in twin pregnancies is the generally slower growth rate observed in all twins during the third trimester and the ongoing debate about whether this represents a pathological process or a benign physiological adaptation [17, 18, 19, 20, 21, 22]. Consequently, there is continued controversy over whether FGR in twin pregnancies should be diagnosed using singleton growth charts, which may classify up to 30%–53% of twin foetuses as potentially growth‐restricted [18, 23, 24, 25], or twin‐specific growth charts, which offer greater specificity [26]. Our finding that mild SGA was associated with an increased risk of abnormal placental pathology only when diagnosed using a twin‐specific chart provides further support for the use of twin‐specific growth charts in the assessment of fetal growth in twin pregnancies.

Another challenge is that, in contrast to singletons, the adequacy of growth in the smaller twin can be assessed not only in relation to published reference growth charts but also by direct comparison with its co‐twin, as reflected by intertwin weight discordance [27, 28]. This approach is theoretically advantageous, as it accounts for the intrinsic growth potential of the twin pair, and may therefore improve the detection of FGR in the smaller twin [28, 30, 31]. Indeed, intertwin weight discordance has been reported as an important risk factor for pregnancy complications, including stillbirth [32, 33, 34] and major neonatal morbidity [32, 34, 35, 36, 37, 38, 39, 40, 55]. However, evidence regarding the optimal discordance threshold and whether discordance is associated with FGR even when the smaller twin is not SGA remains conflicting. In the current study, we found that the probability of abnormal placental pathology increased sharply when intertwin EFW discordance exceeded 20%. Additionally, isolated discordance (i.e., discordance > 20% when neither twin is SGA) was associated with the primary outcome only when SGA was diagnosed using a twin‐specific growth chart, not a singleton chart. Finally, among foetuses with severe SGA, the co‐presence of discordant growth further increased the risk of the primary outcome, suggesting that discordant growth is an independent indicator of placenta‐mediated FGR in cases of severe SGA of the smaller twin. However, it should be emphasised that categorising continuous variables, such as fetal weight percentile and percent discordance, may result in loss of information. Future studies on the diagnosis of FGR in twin (as well as singleton) pregnancies should explore the use of multimodal prediction models that treat predictors as continuous variables, including fetal weight percentile, percent discordance, the exact value of Doppler indices, and the discordance in Doppler indices (such as CPR discordance) [55, 56].

Randomised controlled trial (RCT) data on the optimal timing of delivery in twin pregnancies with suspected FGR are lacking, as twins have been largely excluded from all major RCTs to date on this topic [8, 9, 10]. Consequently, available evidence is limited to observational studies [57], which likely explains the absence of guideline recommendations for the management and timing of delivery in twin pregnancies complicated by FGR [41, 42, 43, 44]. Providing such guidance is especially challenging in cases of late‐onset FGR [7], since current recommendations already support early delivery for all twins, regardless of FGR [41, 42, 43, 44], and it remains unclear whether twin foetuses with late‐onset FGR would benefit from delivery at an even earlier gestational age. A major methodological barrier to designing an effective RCT on the timing of delivery in twins with FGR is the lack of a consistent and validated definition of FGR, in part due to the large number of combinations of diagnostic indicators of impaired fetal growth, such as EFW percentile (based on singleton vs. twin‐specific charts), intertwin size discordance, and abnormal Doppler findings [58]. We propose that our risk categories, which identify homogeneous groups or phenotypes of impaired fetal growth based on the magnitude of risk for placenta‐mediated FGR, can serve as a practical framework to facilitate standardised patient selection and stratification for future randomised trials evaluating targeted interventions and optimal management strategies for twin pregnancies with suspected FGR.

Many prior studies on the diagnosis of FGR in twin (as well as singleton) pregnancies have relied on neonatal outcomes (e.g., low Apgar scores, low umbilical artery pH, admission to the neonatal intensive care unit, or short‐term neonatal morbidity) as proxies for placenta‐mediated FGR [26]. However, the interpretation of these studies is challenging, as many of these neonatal outcomes are not specific to placenta‐mediated FGR and are often influenced by other factors, primarily prematurity. In the present study, we sought to overcome this limitation by using placental pathological findings known to be strongly associated with FGR as the primary outcome, which we considered to be a direct and gestational‐age‐independent indicator of placenta‐mediated FGR that can be used to confirm (or exclude) placental dysfunction as the cause of adverse pregnancy outcomes. Indeed, abnormal placental pathology is strongly associated with fetal growth restriction [59, 60, 61, 62, 63], other placenta‐mediated complications (such as preeclampsia [60, 64, 65, 66] and placental abruption [67]), and adverse perinatal outcomes, including stillbirth [62, 63, 68, 69, 70, 71, 72, 73], neonatal death [62, 74, 75], and neonatal neurodevelopmental impairment [74, 75]. Supporting this rationale, we observed that the severity of FGR was strongly correlated with the risk of abnormal placental pathology, but not with the risk of the secondary composite neonatal outcome. This finding underscores the limitations of neonatal outcomes as surrogates for the postnatal diagnosis of FGR and the need for more accurate and specific biomarkers to diagnose placenta‐mediated FGR in both twin and singleton pregnancies. In addition to abnormal placental histopathology, such biomarkers may include biochemical and angiogenic maternal serum markers [76, 77], as well as molecular placental or fetal markers of hypoxia [78, 79]. While a consensus definition of postnatal FGR has been proposed, its implementation has been hindered by considerable disagreement among the participating experts regarding which parameters should be included in the definition [80]. Moreover, a recent study found that this consensus definition did not improve the identification of newborns at risk of adverse outcomes [81].

### 4.3 Strengths and Limitations

Several limitations of the current study should be acknowledged. First, due to its retrospective observational design, the timing of delivery was likely affected by the severity of FGR as perceived by the managing clinicians. However, as noted above, the primary outcome (abnormal placental pathology) is unlikely to have been influenced by these management decisions. Second, the number of cases of severe SGA, particularly when defined by a twin‐specific growth chart, was relatively small. Still, except for exposure group 5 based on the twin‐specific chart (severe SGA with concordant growth, *n* = 13), the study was sufficiently powered to detect significant associations between most phenotypic groups and the primary outcome. Third, we acknowledge that the number of comparisons (8 exposure groups, 2 outcomes, 2 chart types) increases the risk of Type I error. However, the significant associations in our study were characterised by large effect sizes (aRR > 2.0 – > 4.0) and a biologically logical dose–response relationship, suggesting that the findings likely represent true biological signals of placental dysfunction rather than chance associations. Fourth, it should be noted that mild forms of placental pathologies (such as mild chronic villitis) may be non‐specific and are occasionally observed in uncomplicated pregnancies. However, the relatively low rate of placental pathologies in the low‐risk reference group (Group 1) and the dose–response relationship with worsening ultrasound findings suggest that these placental findings, even when including mild forms, are specific enough to discriminate between pathological and physiological growth. Fifth, our study population was defined based on gestational age at delivery (≥ 32 weeks) rather than the timing of FGR onset. Consequently, the cohort likely includes a subset of foetuses with early‐onset FGR who remained in utero until 32 weeks. While this approach reflects the pragmatic clinical scenario faced by obstetricians in the third trimester, it may introduce heterogeneity regarding the duration and severity of placental dysfunction.

Our study also has several notable strengths. First, we analysed a large and well‐characterised cohort of dichorionic twin pregnancies with comprehensive ultrasound and placental histopathology data. Second, we used a primary outcome that is specific to placenta‐mediated FGR and is unaffected by gestational age at birth, thereby overcoming an important limitation of prior studies. Third, to the best of our knowledge, this is the first study to propose data‐driven risk categories that stratify the incremental risk of underlying placental disease in cases of suspected late‐onset FGR in twin pregnancies.

## 5 Conclusion

In summary, we estimated the risk of placenta‐mediated FGR associated with distinct phenotypes of impaired fetal growth in dichorionic twin pregnancies and proposed risk categories that can be used to estimate the likelihood of placenta‐mediated FGR. This information can refine risk stratification in current practice, for example, by reassuring clinicians that mild SGA diagnosed using singleton charts has a risk of placental pathology comparable to AGA controls. In addition, these findings can inform the design of future trials by providing an evidence‐based framework for selecting homogeneous subgroups of patients with respect to the risk and severity of placenta‐mediated FGR. Furthermore, our findings underscore the potential limitations of relying on neonatal outcomes as a proxy for placenta‐mediated FGR and highlight the need for more accurate and specific biomarkers for the postnatal diagnosis or confirmation of FGR in both twin and singleton pregnancies.

## Author Contributions

Nir Melamed, Ala Essalah and John Kingdom contributed to the conception, design, analysis, interpretation of the data, and drafting of the manuscript. Jon Barrett, Liran Hiersch, and Megan Watson contributed to the interpretation of the data and revision of the manuscript. All authors approved the final version of this manuscript as submitted, and all authors agree to be accountable for all aspects of the work in ensuring that questions related to its accuracy or integrity are appropriately resolved. The guarantor (Nir Melamed) accepts full responsibility for the work and the conduct of the study, had access to the data and controlled the decision to publish. The corresponding author (Nir Melamed) attests that all listed authors meet authorship criteria and that no others meeting the criteria have been omitted.

## Funding

Dr. Melamed holds the Waugh Family Chair in Twin Fetal Medicine Research at the Sunnybrook Health Sciences Centre and the University of Toronto.

## Disclosure

Role of the Funders: The funding bodies played no role in the design and conduct of the study; the collection, management, analysis, or interpretation of the data; the preparation, review, or approval of the manuscript; or the decision to submit the manuscript for publication.

## Ethics Statement

The study has been approved by a Sunnybrook Health Sciences Centre Research Ethics Board #353–2014, Nov‐7‐2024.

## Conflicts of Interest

The authors declare no Conflicts of Interest.

## Supporting information


**Figure S1:** Description of the study cohort.

**Figure S2:** Relationship between fetal weight percentile and intertwin weight discordance with the probability of the primary outcome.
The probability of the primary outcome (abnormal placental pathology) was modelled as a function of estimated fetal weight percentile (A) and intertwin estimated fetal weight discordance (B) using restricted cubic splines. For estimated fetal weight percentile, the relationship is presented for percentiles calculated using a singleton chart (blue line) and a twin‐specific chart (red line).
EFW, estimated fetal weight.

**Figure S3:** Rate of the specific types of placental pathology by the exposure group.
The rates of MVM pathology (orange line), FVM pathology (green line) and chronic villitis (blue line) are presented for each phenotype of impaired fetal growth, defined using a singleton chart.
SGA, small for gestational age; AGA, appropriate for gestational age; MVM, maternal vascular malperfusion; FVM, fetal vascular malperfusion.

## Data Availability

The data that support the findings of this study are available on request from the corresponding author. The data are not publicly available due to privacy or ethical restrictions.
