# Supplementary figures and images for: Risk of Placental Pathology Across Ultrasound‐Defined Phenotypes of Impaired Fetal Growth in Dichorionic Twins: A Retrospective Cohort Study
Source: BJOG. 2026 Mar 11;133(8):1616–28. doi: 10.1111/1471-0528.70215 (PMC13254041; doi:10.1111/1471-0528.70215)

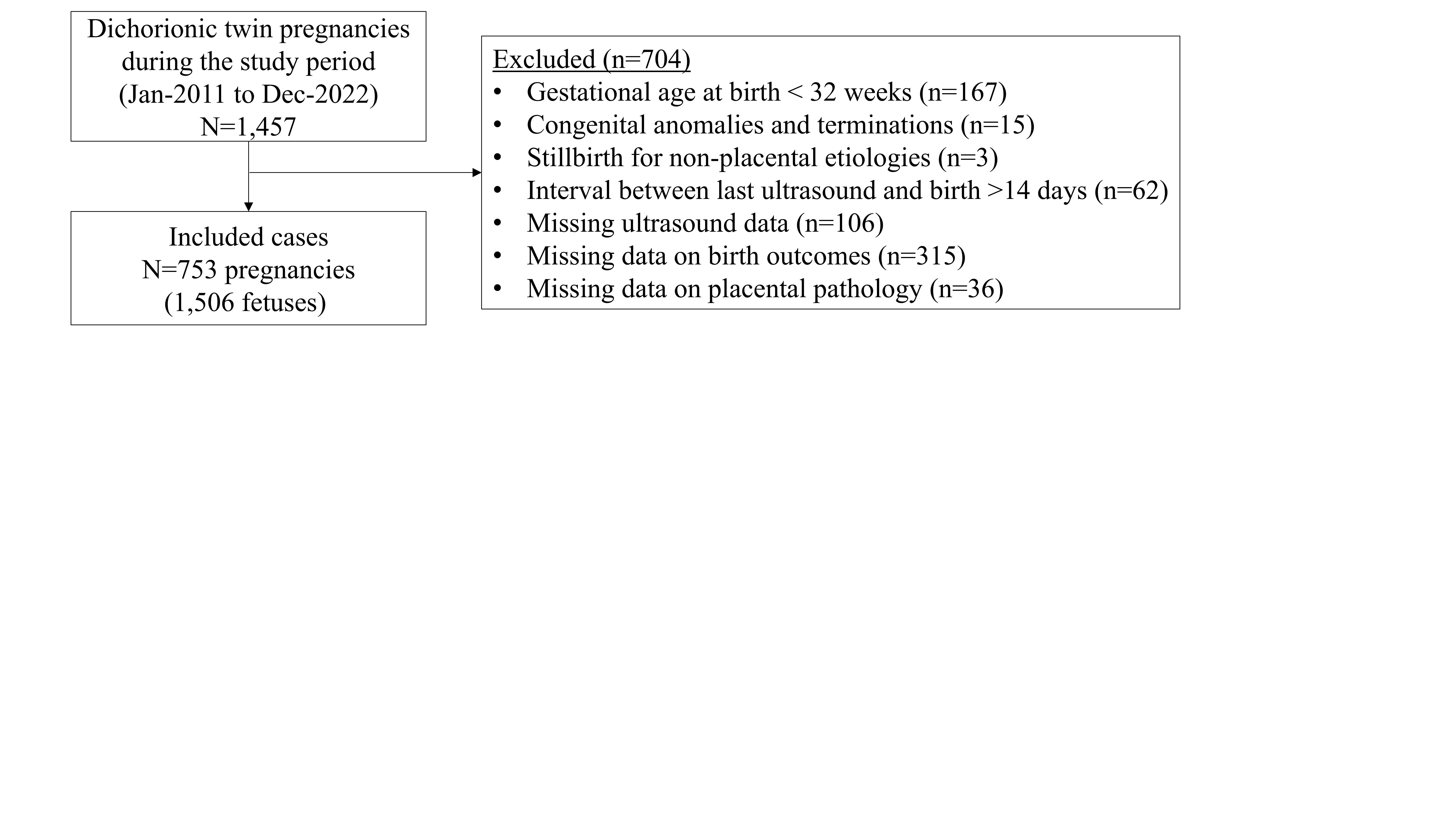

Supplement: Supplementary file 1 — Figure S1: Description of the study cohort. [file BJO-133-1616-s002.tif]

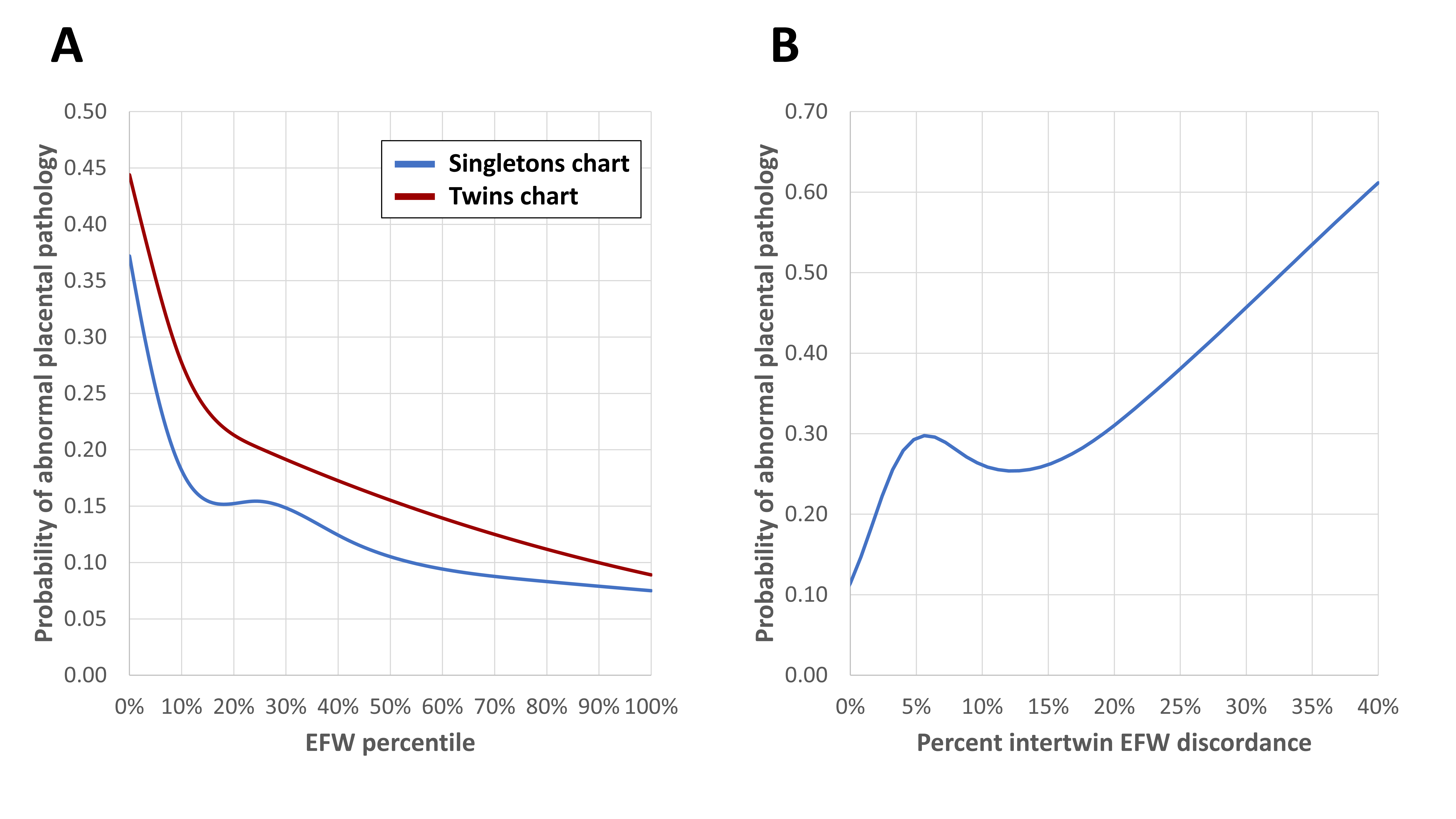

Supplement: Supplementary file 2 — Figure S2: Relationship between fetal weight percentile and intertwin weight discordance with the probability of the primary outcome. The probability of the primary outcome (abnormal placental pathology) was modelled as a function of estimated fetal weight percentile (A) and intertwin estimated fetal weight discordance (B) using restricted cubic splines. For estimated fetal weight percentile, the relationship is presented for percentiles calculated using a singleton chart (blue line) and a twin‐specific chart (red line). EFW, estimated fetal weight. [file BJO-133-1616-s003.tif]

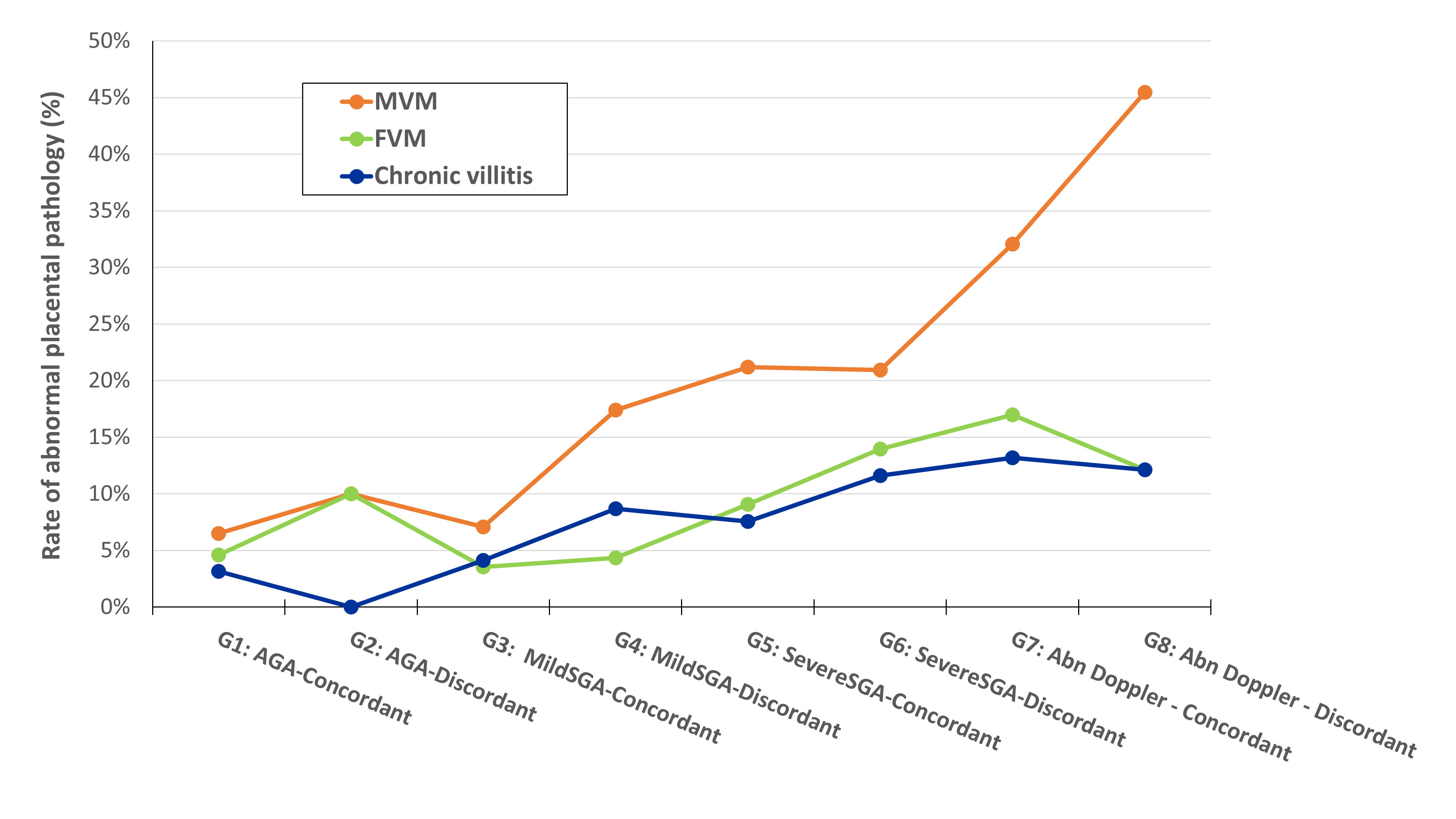

Supplement: Supplementary file 3 — Figure S3: Rate of the specific types of placental pathology by the exposure group. The rates of MVM pathology (orange line), FVM pathology (green line) and chronic villitis (blue line) are presented for each phenotype of impaired fetal growth, defined using a singleton chart. SGA, small for gestational age; AGA, appropriate for gestational age; MVM, maternal vascular malperfusion; FVM, fetal vascular malperfusion. [file BJO-133-1616-s001.tif]
